# Remote Real-Time Monitoring of Subsurface Landfill Gas Migration

**DOI:** 10.3390/s110706603

**Published:** 2011-06-27

**Authors:** Cormac Fay, Aiden R. Doherty, Stephen Beirne, Fiachra Collins, Colum Foley, John Healy, Breda M. Kiernan, Hyowon Lee, Damien Maher, Dylan Orpen, Thomas Phelan, Zhengwei Qiu, Kirk Zhang, Cathal Gurrin, Brian Corcoran, Noel E. O’Connor, Alan F. Smeaton, Dermot Diamond

**Affiliations:** CLARITY, Centre for Sensor Web Technologies, Dublin City University, Glasnevin, Dublin 9, Ireland; E-Mails: cormac.fay@dcu.ie (C.F.); aiden.doherty@dcu.ie (A.R.D.); stephen.beirne@dcu.ie (S.B.); fiachra.collins@dcu.ie (F.C.); Colum.Foley@computing.dcu.ie (C.F.); john.healy2@mail.dcu.ie (J.H.); Breda.Kiernan@dcu.ie (B.M.K.); hlee@computing.dcu.ie (H.L.); damien.maher@dcu.ie (D.M.); dylan.orpen@dcu.ie (D.O.); thomas.phelan4@mail.dcu.ie (T.P.); zhengwei.qui@computing.dcu.ie (Z.Q.); ke.zhang@ucd.ie (K.Z.); cgurrin@computing.dcu.ie (C.G.); Brian.Corcoran@dcu.ie (B.C.); Noel.OConnor@dcu.ie (N.E.O.)

**Keywords:** environmental monitoring, greenhouse gases, chemistry, landfill, carbon dioxide, methane, sensor networks, sensor data management

## Abstract

The cost of monitoring greenhouse gas emissions from landfill sites is of major concern for regulatory authorities. The current monitoring procedure is recognised as labour intensive, requiring agency inspectors to physically travel to perimeter borehole wells in rough terrain and manually measure gas concentration levels with expensive hand-held instrumentation. In this article we present a cost-effective and efficient system for remotely monitoring landfill subsurface migration of methane and carbon dioxide concentration levels. Based purely on an autonomous sensing architecture, the proposed sensing platform was capable of performing complex analytical measurements *in situ* and successfully communicating the data remotely to a cloud database. A web tool was developed to present the sensed data to relevant stakeholders. We report our experiences in deploying such an approach in the field over a period of approximately 16 months.

## 1. Introduction

### 1.1. Global Environment

Global warming is recognised as a serious worldwide challenge. The Intergovernmental Panel on Climate Change (IPCC) fourth report states that the warming of our climate is evident and that human activities are very likely the cause through the emission of substantial amounts of greenhouse gases into the atmosphere [1]. In this article we focus on greenhouse gases (methane and carbon dioxide) emitted from the decomposition of biodegradable waste at landfill sites.

### 1.2. Chemical Sensing and Information Retrieval from the Environment

It is well documented that the quality of our environment is determined by its chemistry, and imbalances in a wide variety of parameters can have a drastic effect on air and water quality, leading to increases in the incidence of respiratory diseases and cancers, amongst others [2–5]. Our basic sensing capabilities are limited and only allow us to observe the aftermath of the effects that these substances ultimately cause. The ability to harvest chemical information from our environment can provide the means to enforce preventative measures and/or to provide early warning. Hence, chemical sensing is on the increase and is very much encouraged by local and global governmental legislation, such as the Water Framework Directive [6], Kyoto protocol [7], Climate Change Act [8], Global Warming Solutions Act [9].

However, target-specific environmental chemical sensing is not easy to achieve due to the many problems of integrating chemical sensors into practical, long term sensing platforms [10]. For instance, to realise a fully functional chemical sensing system, the designers must face a multitude of multidisciplinary issues such as: reduction of the sensing surface, drift, cross sensitivity, bio-fouling, chemo-electronic transducer, power consumption, elemental robustness, security, vandalism/damage, autonomous control, successful and secure delivery of data to stakeholders/authorities.

The concept of Internet Scale Sensing (ISS) for chemical sensing is well known but it should be noted that although new and emerging techniques from the digital and chemical worlds continue to progress, a critical missing element is “the gateway” linking these two realms [11]. A key goal for autonomous environmental monitoring is in providing the ability to easily access that data by relevant stakeholders such as environmental enforcement agencies. It is believed that the use of the *“cloud computing”* concept, where data is stored on a server and is always available via the Internet, will be the means to access harvested environmental data. This data can be viewed across a wide variety of computing/software platforms, e.g., internet browsers, iPhones, iPads, smart phones, *etc*. The final vital ingredient behind the Internet Scale Sensing vision is in turning the massive volume of raw sensor data into meaningful information. Some have suggested that this process will be achieved via intelligent signal processing with event detection models [12]. After those events have been identified, software outlier detection algorithms can then be employed to identify the events of most interest and subsequently alert the relevant stakeholder(s).

### 1.3. Landfill Emissions and the Current Monitoring Standard

Waste activities account for approximately 5% of the global greenhouse gas (GHG) emissions and of all the waste management methods in use, land-filling is by far the most common [13,14]. The major components of landfill gas (LFG) are typically carbon dioxide (CO_2_: 40%–60%) and methane (CH_4_: 40%–60%) from the decomposition of biodegradable waste [15,16].

During landfill cell development, and before introduction of the waste body, the surrounding soil is covered with a mineral layer in conjunction with a geosynthetic liner. Further to this, a network of perforated pipes is implemented in order to fully extract the landfill gas being produced. After capping, the gas is subsequently treated and/or disposed of in a safe manner through flaring or venting [17].

Following decomposition of the waste, landfill gas generation can begin as early as 6–12 months after capping and typically continue for a further 20–50 years [18] and even as long as several hundred years in some cases [19]. In addition, serious health issues have been linked to landfill site proximity [20–23]. It is clear that limits must be put in place on the gas concentrations emissions. As a result, the waste license for landfill sites, from the Irish Environmental Protection Agency, states that concentration levels (measured from perimeter borehole wells) must not exceed 1% for methane and 1.5% for carbon dioxide [24] (it is noteworthy that the lower explosive limit for methane is 5% v/v in air). To comply with these levels, the site’s flare is designed to dispose of harmful gases (e.g., methane) through the gas extraction network. If a problem is identified (through manual monthly monitoring) such as an increasing gas concentrations, the flare is then set to increase its production.

Arising from legislative enforcement, emission levels from landfill sites require continuous monitoring including sub surface migration of gases [25]. Monitoring is required to take place at an average frequency of once per month—and can even be as infrequent as 4 times per year in some cases—by using a hand-held gas analyser to monitor samples extracted from the top of perimeter borehole wells for a duration of approximately one minute. Subsequently, these levels are manually reported to national environmental protection agencies by the license holders via email/phone. If in breach of permitted gas concentration limits, fines are subsequently levied. However elements of human error and variability across operators can provide problems. Also, in extreme situations, this process is vulnerable to manipulation because inspectors must announce their visits beforehand, allowing for the possibility of high concentration gases to be vented to the atmosphere prior to inspection.

Most of the available gas analysers suitable for landfill gas monitoring are handheld based. At present, a majority of the borehole wells in Ireland are monitored using the GA2000 (Geotech) as standard. However other available products offer similar functionality including GA90/GEM500/GEM2000 (Landtec), G3 LMSxi (Ashtead) and Gas-Tec (AFC). In the same manner, specialised instruments exist that offer a continuous monitoring feature suitable for landfill site monitoring including IR600 (Hitech Instruments), LFG2003 (Liberty Engineering), Ultramate 23 (Siemens), AEMS (Geotech) *etc*. However, many of these systems are heavy and relatively expensive, require specialised personnel to maintain and install, demand high power (most requiring mains power) and are aimed at monitoring at the flare only *i.e.*, giving a global reading for the entire extraction system network. Hence, there is a need for portable, low power, low cost instrumentation to continually monitor localised areas and ultimately replace the manual monitoring tasks.

### 1.4. Chemical Sensing of Greenhouse Gases from Landfill Sites

Given this background we feel that an automated monitoring technique is essential to reduce the cost of manual sampling, improve the reliability of the gas concentration levels reported to governing authorities and ultimately provide a means to better identify elevated GHG emissions, which may lead to additional steps to reduce those emissions.

In this paper we apply the template of internet scale sensing and detail a prototype end-to-end sensing model for landfill emission monitoring, which has been deployed in field studies over a time period of 16 months across three different locations. We have collected 432,540 sensor readings with 2,403 remote communications back to our web database. Specifically, we describe the gateway platform (Section 2), show approximately one year of harvested data from an extended *in-situ* trial on an active landfill site (Section 4.3) and finally, we describe potential improvements which can be implemented from this development phase to further reduce costs (Section 4.6).

## 2. Internet Scale Sensing for Landfill Emission Monitoring

### 2.1. Sensing Model

To achieve our goals, we applied the concept of internet scale sensing (ISS) and expanded it to suit a realised sensing model for real-time landfill migration monitoring via a live web-site.

**Figure 1.**
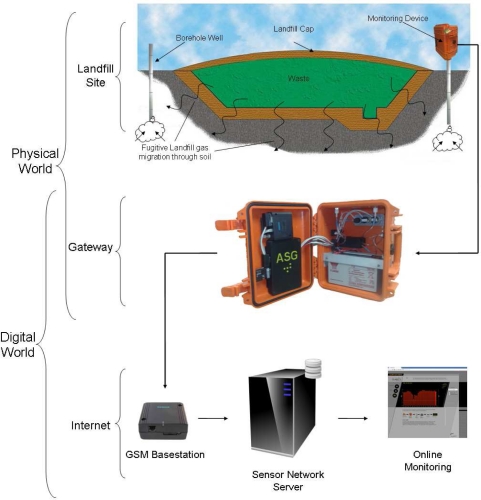
Visual representation of the landfill gas sensing model from the device placed in the field to a web-based visualisation user interface. The model shows the progression of chemical sensed data from the physical world to the digital world by means of a gateway platform.

Figure 1 provides a visual representation of our end-to-end sensing model. Firstly, the physical/chemical gas sensors are exposed to the sample landfill gas and generate an electrical signasl that are proportional to the concentration of the constituents within the target gas sample. These signal lines are conditioned by the gateway platform and digitised by the system’s micro-controller. At this point, the sensor data are stored on-board and transmitted via the GSM network to a base station where the data are then forwarded to a web-enabled sensor network server. After storage on a relational database, the data are visually presented to web users and governing authorities by means of an easy to interpret web page. The following sub-sections gives a more detailed explanation of these processes.

### 2.2. Gas Sensors

The system was equipped with a humidity sensor (Honeywell HIH-4000-001), linear range 0 to 100% RH, and a temperature sensor (a 10 kΩ thermistor—Thermometrics DKF103N5) with an operational response range of −40 °C to +250 °C. Both temperature and humidity measurements are critical for providing background information when analysing gas samples, especially during developmental stages and for correlation with environmental artifacts that may give rise to erroneous signals from the chemical sensors.

More importantly, the system was also equipped with two chemical gas sensors; for CH_4_ and CO_2_ detection. As discussed earlier (Section 1.3), landfill gas is composed mainly (greater than 95% [16]) of CH_4_ and CO_2_, thus these were the principle sensing targets. Although the permitted concentration limits of both gases are low (see Section 1.3), it was found through previous developmental phases and field validation trials that measured readings were up to 10 times that of the allowed regulated range [26]. As a result, both sensors (NDIR based) were sourced from Dynament Ltd. (www.dynament.com) with a custom range of 0%–20% to suit problematic landfill sites. These sensors were designed to be self compensating to both temperature and humidity in the ranges of −20 °C to +50 °C and 0% to 95% RH respectively [27,28].

### 2.3. Gateway Platform

#### System Components

**Figure 2.**
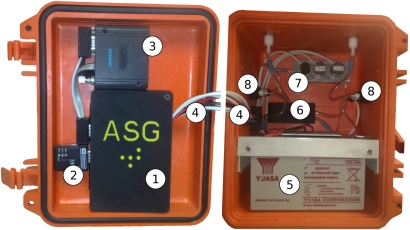
Component layout of the gateway platform. (1) control system, (2) bluetooth module, (3) GSM module, (4) signal and actuation control lines, (5) power source, (6) extraction air pump, (7) gas chamber, (8) flow selection valves.

All system components were housed within a robust case (Peli Case 1300). An internal volume of 25.1 (L) × 17.8 (W) × 15.5 (H) cm allowed sufficient space for all system elements to be securely packaged and for a systematic layout of components. The case’s lid held the most accessible and frequently used system elements employed by users during system development and deployment, *i.e.*, control and communications. Conversely, the sensors and actuators were positioned within the case’s base for secure and fixed positioning while allowing sufficient room for gas tubing and electrical connections. Figure 2 shows the layout views of the system’s components for control/communications and sensors/actuators respectively.

1. Micro-controller board
  A custom, in-house designed and assembled PCB (Printed Circuit Board—manufactured by Beta LAYOUT Ltd.) was developed to suit the requirements of this project. At its core lay an MSP430F449 (Texas Instruments) which controlled component power switching, timing, signal conversion, data handling, storage, communications *etc. i.e.*, all processes necessary to achieve full autonomy of the system.
  The board was equipped with ten switch-able power ports (8 × PFETs and 2 × NFETs). Each port’s power supply was capable of being manually selected (via on board jumper pins) from 3 separate voltage supplies *i.e.*, 3V3 regulator (Texas Instruments, LP2985A), 5 V regulator (National Semiconductor, LP2992IM5) and 12 V (main battery).
2. Short range communications
  A miniature bluetooth module (LM Technologies LM048) was included for local, short range communications. This feature allowed users to interface with the system without exposing the electrical components/connections such as the microcontroller to external conditions, such as rain. The module communicated to the microcontroller through a DE9 connection, via an RS232 transceiver (Maxim, MAX3232CSE) and finally to one of its UART channels. Power to the bluetooth module was applied by means of an external, weatherproof (IP67 rated) switch and using the 5 V voltage regulator.
3. Long range communications
  Remote reporting of landfill gas concentrations to a local base station was achieved by means of a GSM module (Siemens MC35iT). This allowed stand-alone developmental systems to be deployed in very remote locations while still being able to communicate data back to stakeholders. Power was supplied via three PFET power switches. Communications were achieved in a similar fashion as with the bluetooth module with the exception of using the microcontroller’s secondary UART channel.
4. Signal and actuation control lines
  Wiring for switching power to components was of single core and insulated (RS 140420). Sensor signal lines were connected using shielded wiring (RS 1643740).
5. Power source
  A low cost, rechargeable and high capacity 12 V battery supplied power to the entire system (YUASA-NP712). Note: a 2× AAA battery pack supplied power to the microcontroller (behind a diode) for an uninterrupted power supply to the microcontroller which allowed the main battery to be changed with no loss of RAM e.g., the real time clock.
6. Extraction pump
  An air pump (SKC Grab Air 222–2301) with closed circuit input/output ports allowed sample gas to flow through the sample chamber at a rate of 0.6 L/min. This was actuated by a single microcontroller I/O port and one NFET with power from a 5 V regulator.
7. Gas chamber
  A custom designed gas chamber accommodated all sensors and two port connections. It was fabricated using a Dimension SST 768 rapid prototyper and uniformly sealed using a combination of MEK and silicon coupled with O-rings for sensor access.
8. Flow selection valves
  In order to select the supply source and target exhaust flows, we equipped the system with two 3/2 way latching pneumatic control valves (Lee Products Ltd. LHLA0531211H).

#### Sampling Procedure

**Figure 3.**
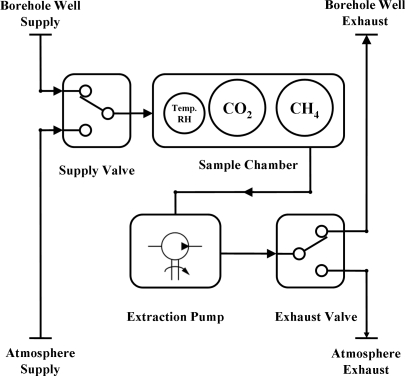
Schematic flow diagram illustrating the gas flow control system. The flow control valves allow the system to be switched between sampling mode (from ‘Borehole Well Supply Supply’ to the ‘Borehole Well Exhaust’) and baseline and purge modes (from ‘Atmosphere Supply’ to the ‘Atmosphere Exhaust’)

As discussed earlier, the existing legislative monitoring procedure (for determining landfill gas emissions from perimeter borehole wells) calls for a sampling frequency of once per month, extracting gas for circa 1 minute using a hand-held instrument and venting to atmosphere (Section 1.3). In this study, we increased this sampling frequency 60 fold. However, this immediately meant that much more greenhouse gases would be emitted to atmosphere when following the existing sampling procedure; a contamination that this device was ultimately expected to reduce. To address this issue, a previous study was conducted to investigate a recycling technique whereby the sampled gas was exhausted back into the borehole well (at a different depth) instead of venting to atmosphere [26]. The findings of the study showed that this recycling technique (of landfill gas recycling back to the borehole well) did not affect the gas composition measurements when compared with venting to atmosphere. Moreover, the same study discovered that when multiple borehole wells of various head space depths were sampled, the longest time to achieve a steady state measurement was circa 2 minutes. To allow for an appropriate settling time, a 3 minutes monitoring duration was chosen. As a result, the automated monitoring time was divided into 3 separate procedures (*baseline*, *sample* and *purge*) where each was monitored for a duration of 3 minutes and sampled at 3 seconds intervals. The device’s air flow control system is illustrated in Figure 3.

Firstly, for the *baseline* procedure, the “Supply Valve” was switched to the “Atmosphere Supply”, and the “Exhaust Valve” to the ‘Atmosphere Exhaust’ settings. The motivation for this step was threefold. The primary reason was to check that the sensors were in fact powered up, *i.e.*, a valid measurement gave a value of no less than the sensors standard offset of 0.4 V at 0% v/v (if unpowered the sensors report an electrical potential of 0 V). Coupled with this, it allowed sufficient time for the IR sensors to warm up and stabilize; a necessary step for accurate measurements. Furthermore, it was also ensured that no residual landfill gas was present in the chamber from previous measurement cycles. Subsequently, the *sample* procedure took place whereby both valves were toggled from the *baseline* step so that landfill gas was extracted from the “Borehole Well Supply” and exhausted to the “Borehole Well Exhaust”. Finally, the valves were again toggled to the state used for the *baseline* procedure. The following *purge* procedure was used to remove the landfill gas from the gas chamber.

#### Signal and Data Flow

A total of five signal lines were connected to the microcontroller’s analogue to digital converter (ADC) channels. As a rule, the ADC channels may only digitise voltage levels between 0 V and 3.3 V. The dynamic range of each analogue signal line was first conditioned to lie within the ADC measurement range by means of appropriate signal conditioning circuitry. Once conditioned, the sensor outputs were digitised, via the ADC channels, at 3 seconds intervals over the full 9 minutes sampling procedure. Along with the sensor data, a single battery reading was saved as part of the data set, along with a timestamp according to the on board real time clock (set to GMT). The full data set was retained in RAM before storage or reporting back to the stakeholder. Following this, the data was saved as raw ADC values on two separate 2 Mbit flash chips (Numonyx M25P20-VMN6P) allowing a total of 131.072 Kbytes to be stored over 8 separate sectors. The data were arranged in a structured format to utilise all data bits in each byte. The first, second and third sectors on each chip were assigned to store the data harvested during the baseline, sample and purge procedures respectively. By the same token, the fourth sectors were kept in reserve for additional sensor data overflow or system specific settings. As a result, the system was able to save up to two years of separate trial data assuming a sampling frequency of twice per day.

#### Communications

As environmental devices are often placed in remote locations, one of the only readily available methods of wirelessly transmitting sensed data to stakeholders is to take advantage of the GSM network’s national coverage. The system’s GSM module was powered via three of the eight positive switching power channels for mains power, wake up and shut down triggering. After harvesting and storing landfill gas data from a monitoring cycle, a statistical representation was compiled (average, max, min) and sent to the Sensor Network Server (SNS). SMS messaging is used at present with optional use of the module’s GPRS feature; however it was found to be more effective to use SMS for maintenance and continuous power costs. For complete dataset retrieval, one must interface with the device and download stored data from the on-board flash memory chips. Initially, this was achieved in the same manner as in a laboratory setting, *i.e.*, a direct wired connection to a laptop PC. However, this method is highly undesirable (*i.e.*, to open the system *in situ*) as it left the system vulnerable to such conditions as rain, wind and biofouling, potentially causing damage such as electrical shorting. As a result, the system was equipped with an external power control switch (IP67 rated) for the Bluetooth module. Thus, full system control was accessible for diagnosis of individual components, setting the system clock and retrieval of data without breaching the system’s environmental seal.

### 2.4. Sensor Network Server

#### Base Station and Database Interface

**Figure 4.**
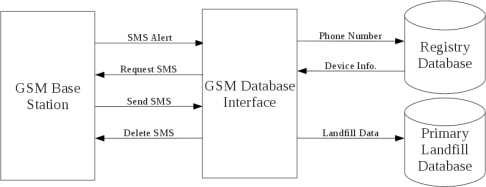
Block diagram showing the interactions between the GSM base station, the GSM database interface and the relational databases. The remotely reported data is received by the GSM base station where, through a number of programming stages, the data is stored on the primary landfill database.

The GSM base station (Siemens MC35iT) was connected to a server PC via a RS232 interface (Rotronic UC232A) and powered by a standard 12V power adapter (Masterplug MVA1200-MP). Written in the Java programming language and using the Javax.comm package, a custom written application, denoted here as the GSM Database Interface (GDI), provided the functionality to progress data from new Landfill SMS texts to a secure MySQL database. Figure 4 depicts the overall process of advancing the data from the GSM base station to the primary environmental database.

At first, the base station was placed into “SMS Alert Mode” where, upon receipt of a new SMS, a string of characters was sent to the server indicating which slot the new text was stored within the GSM’s memory. Once the GDI was alerted to a new packet, it automatically parsed the alert string and extracted the SMS memory storage address on the base station. Next, the GDI requested and received the full SMS text located at the appropriate memory address. After parsing the new text header, the sender’s phone number was used as the unit identification factor. At this point the GDI connected to a “registry database” where it received all relevant information related to the remote device such as type, unit number and calibration settings based on the sender’s phone number. Next, the text body was parsed. All reported measurements were extracted and automatically placed on the database using standard SQL statements. Also, inline conversions of ADC to CO_2_, CH_4_ and battery levels occurred at this point using previously acquired calibration data (Section 3.1) in the lab. Finally, the text was deleted from the base station as the storage capacity of the SIM card was limited.

#### Data Organization

**Figure 5.**
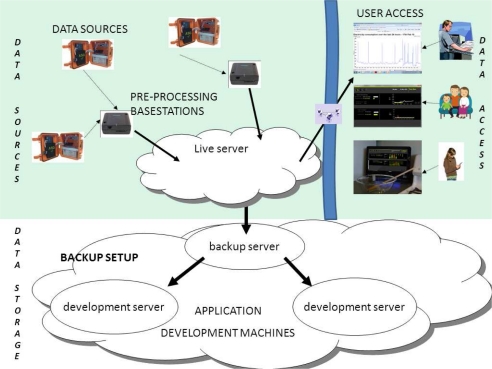
Overview of Data Storage, Backup and Presentation. Multiple landfill sensors can upload data to a single cell base-station. Thereafter these base-stations upload data to a central server, which is also backed up. Finally this data is available via the Internet for end users to access.

Once data was transmitted via the GSM network to a central base-station, the data was stored in a relational database which can manage data from multiple landfill locations. This relational database was constantly available on the Internet which thus fulfils our mission of data accessibility, and also it was stored in a replicated database setup; meaning that there was multiple copies of the data, thus ensuring data redundancy. Figure 5 provides an overview of our end-to-end system, from sensing our environment through to informing users of the relevant levels of CO_2_ and CH_4_ in their local landfill site.

#### Data Presentation

**Figure 6.**
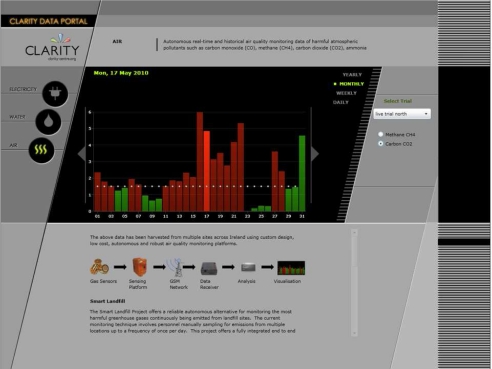
Sensor Data Portal: Our web application which allows relevant stakeholders to easily view in real-time the air quality (CO_2_ & CH_4_) data from landfill sites. The website can be viewed at http://clarityapp.ucd.ie/~sensorportal/.

The data were accessible by relevant stakeholders via an intuitive web interface in which the data were displayed graphically. Figure 6 shows the designed Silverlight web interface. Initially the user is presented with a list of trials (both live and historical) via a combo box on the right hand side of the screen. After selection of a relevant trial the user can click on the relevant radio button on the right hand side to view either the CO_2_ or CH_4_ values, which are displayed as a bar chart, with yellow-green bars indicating values within recommended limits and orange bars indicating that the limits have been exceeded. The user can change the visualisation unit to daily, monthly, and yearly by clicking on the option buttons, and arrow buttons are provided to slide the time period to the previous or next time span. Finally, below the main display area a brief description of how the information is collected is illustrated.

## 3. Methodology

In this section we now describe the various procedures and experimental setups that were utilised to evaluate our system. Firstly, it was necessary to thoroughly calibrate the relevant sensors in a laboratory environment. Most importantly, we then describe our protocol for field deployment for a period of over one year. Finally, we review the protocol to carry out post-analysis software optimisation simulations on the array of data gathered in the field in the previous year.

### 3.1. Calibration of Chemical Sensors

Before any deployment of the system could take place it was first essential to calibrate the chemical sensors. At first, the system was setup in the same configuration for field trials *i.e.*, when sampling and exhausting to/from a borehole well except the supply was connected to source gases and the exhaust to a fume hood extraction system. Next, the microcontroller executed a pre-programmed calibration routine where all the sensors were powered on and the digitised ADC values were continually output (in an endless loop at a frequency of 0.33 Hz) to a laptop computer via a serial port and captured using Microsoft Hyperterminal (a command line interface communications utility).

Each sensor was calibrated against source gases (CO_2_/CH_4_ supplied by Scott Specialty Gases) at concentrations of 0% to 50% with a nitrogen balance. Coupled with a dilution of ambient air, sourced using an air compressor (Werther International 42040 100/50), and managed with mass flow controllers (Cole Parmer YO-32708-26), various gas concentrations were achieved for calibration of the CO_2_ and CH_4_ sensors. A flow rate of 0.6 L/min (matching the flow rate of the system’s air pump) was ensured using a standard flow meter. Furthermore, a GA2000 Plus device (the current landfill gas monitoring standard hand-held gas analyser) was used as reference and verification of both gas concentrations and flow rate. A ten point calibration plot for each IR sensor (CO_2_ and CH_4_), was achieved (Section 4.1).

### 3.2. Power Usage

The system was programmed to autonomously wake up from a low power mode every 12 hours (changed to every 6 hours subsequently), perform a monitoring (analyse gas composition and report) cycle and subsequently return to its low power state. As this system was placed at a remote location, it was desirable to establish how long the system would remain operational using the existing power source (7 Ah lead acid battery). Consequently, current consumption analysis was performed using a high end multi meter (Keithly 2700) capable of sampling at a frequency of 60 Hz with a resolution of 9 decimal places. The landfill system’s power source was connected in series with the multi meter and configured for a typical monitoring cycle—see Section 4.2 for analysis.

### 3.3. System Deployments

While the overall sensing model was in the final stages of development, including the implementation of 3 deployable platforms, a parallel effort was undertaken to find a suitable location for our first deployment. Ideally we wanted a site that would allow us access, be safe from possible vandalism and have an eventful borehole well to monitor. As many landfill sites in Ireland are privately owned, our first criteria was not easily met. After much evaluation, we located a site (closed to public access) where the personnel were very accommodating and enthusiastic, as they had a problematic well that needed continuous monitoring (*location A*). After the site personnel experienced the remote sampling advantages of the system, we were asked to monitor a second well on the same site (*location B*). Some time afterwards, our national environmental enforcement agency requested that we deploy another system to a problematic site that they were dealing with at the time (a borehole well with high concentrations of CO_2_), (*location C*). Stakeholder involvement is time-consuming in building up a working relationship, but critical to the success of research efforts like this.

After calibrating our sensors we were then ready to deploy 3 landfill systems into the physical environment. From our experience with previous developmental models [26,29,30], we were confident that the systems were sufficiently robust to withstand long term deployments in the environment. Initially we deployed one landfill unit to *location A* on the 28th May 2009, which sampled twice a day (11 AM + 11 PM) until the 8th October 2009. From the 13th August 2009 we then deployed a second landfill unit to *location B* to concurrently sample twice a day (11 AM + 11 PM) (Our field-trial deployments are listed in Table 1, and will be explained in more detail in Section 4.3). After these two trials, we recalled the units to our research labs to carry out some maintenance work after finding that insects had breached the system during a regular battery change. At this point, the systems were thoroughly examined, cleaned and the PCB boards were protected by a layer of spray silicone (Electrolube ERDCA200H). Between November and December we redeployed one landfill unit to *location A*. After further maintenance, from early March 2010 until the present time we have been sampling at 4 times every day (12 AM, 6 AM, 12 PM, 6 PM) in both *location A* and *location B*. From May 24th we added a third location, *location C*, meaning 3 locations were being sampled 4 times per day at 12 AM, 6 AM, 12 PM, and 6 PM.

### 3.4. Deployment Data Processing

As described earlier, each monitoring cycle consisted of a 3 minutes *baseline* (60 samples taken at 0.33 Hz), a 3 minutes *sample* (60 samples taken at 0.33 Hz), and a 3 minutes *purge* (60 samples taken at 0.33 Hz), with a statistical representation of each of the 3 stages being sent back to our central base-station via GSM. Meanwhile the fully recorded dataset was stored in on-board flash memory in the landfill system, and then downloaded at a later date for further analysis. We then had the ability to take this data back to our research labs (downloaded during battery changes) to carry out post-event analysis and determine what the optimum sampling rate should be for future deployments of our landfill systems. The objective of this exercise was to improve battery lifetime, and reduce transmission and processing costs.

To carry out our computational analysis we considered data from *location A* between 29th July 2009 and 9th October 2009. This equated to 143 9-minute monitoring cycles (baseline + sample + purge) taken over this period of time. In this exercise, we considered the raw sensor data (recorded every 3 seconds) which equated to 25,740 readings. A software processing algorithm was then used to go through 95 scenarios on all 143 × 60 baseline/sample/purge readings, thus representing optimisation investigations on 2,445,300 simulated data readings. In the results section we will report on our findings as to the optimal sampling rate.

As noted in Table 1 there was a period of time in our *location C* deployment where there was a 9 minutes sampling period to/from the borehole well (with no baseline extraction beforehand or purge extraction afterwards). These 93 instances (16,020 raw data samples) allowed us the opportunity to investigate whether the nature of the sample stage remains similar without baseline and purge stages. This could potentially allow for many savings in terms of shorter sampling times (power, memory and communications loads) and being able to disregard valves (component cost, complexity).

## 4. Results and Discussion

### 4.1. Chemical Sensors

**Figure 7.**
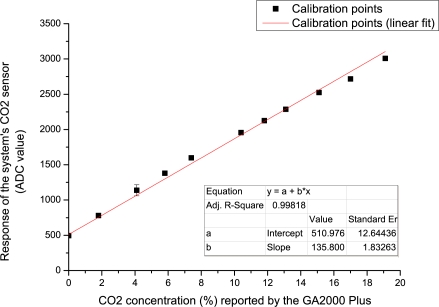
Calibration of the system’s CO_2_ infrared gas sensor. Points represent the average of the steady state response over circa 2 minutes. Error bars (present but difficult to see due to the high sensor accuracy) represent the standard deviation.

**Figure 8.**
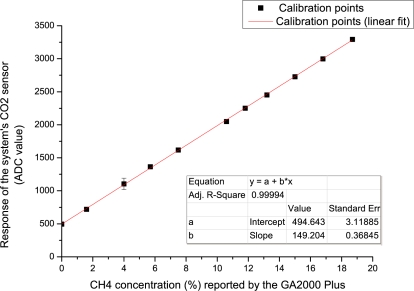
Calibration of the system’s CH_4_ infrared gas sensor. Points represent the average of the steady state response over circa 2 minutes. Error bars (present but difficult to see due to the high sensor accuracy) represent the standard deviation.

The calibration routine of the chemical sensors was described earlier (Section 3.1). At each of the 10 calibration steps we extracted the data when each sensor arrived at a steady state response. Once this was achieved, we noted the respective gas concentration levels from our reference instrument (GA2000 Plus). A 10 point calibration plot for each IR sensor, CO_2_ and CH_4_, is presented in Figures 7 and 8, respectively. Excellent correlation between the reference system and landfill sensors was obtained for CO_2_ (R^2^ = 0.99818, n = 10), and for CH_4_ (R^2^ = 0.99994, n = 10). It is clear that the system’s detection performance is on par with the currently used reference instrument when detecting these two chemical targets. Finally, the linear correlation equations generated by these calibration plots were used as inline conversions from reported ADC measurements by the remote systems for presenting real concentration values online.

### 4.2. Power Consumption Analysis

**Figure 9.**
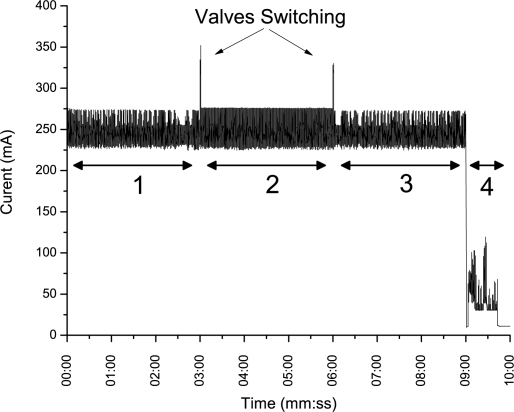
Current analysis of the landfill system during a full monitoring routine (1) baseline procedure, (2) sampling procedure, (3) purge procedure, (4) communications and storage procedure.

Figure 9 shows the current consumption analysis during an aforementioned monitoring and reporting routine (Section 3.2). One can see identifiable trends relating to the four procedures (baseline, sample, purge and communications), and also the times when the extraction and exhaust valves were toggled. The average current consumption during this time was found to be circa 230.1 mA over a duration of 9 minutes and 45 seconds (585 seconds in total). By the same analysis method, when in its inactive state, the multimeter reported an average current draw of 6.13 mA for 42,615 seconds before the next sampling routine. This was calculated to be an average continuous current draw, from the 12 V source, of 9.16 mA. Assuming an ideal power source with these characteristics, the system (with its present power source and sampling routine) can autonomously monitor landfill gas concentrations for an estimated 4.5 weeks which was a sufficient deployment time (without requiring a battery change) to explore this proof of principle study.

### 4.3. Deployment Data

**Figure 10.**
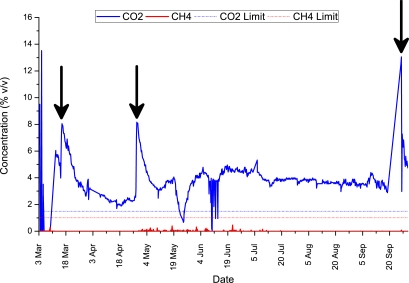
CO_2_ and CH_4_ readings from a 7 month field deployment at *location C* between March 2010 and October 2010. Note that CO_2_ exceeds the recommended limit 96.6% of the time, while CH_4_ never exceeds the recommended limit. The arrows on the graph illustrate significant CO_2_ events that were recorded around the 17th of March, 28th April, and 25th of September. There were no CH_4_ events.

Table 1 summarises the collection of data over a 16 month period as described in Section 3.3. Overall, we can observe that 2,403 samples were sent to the central server, in which the CO_2_ limit was exceeded in 1,490 (62%) samples! The CH_4_ limit was exceeded on 190 (8%) occasions, while both CO_2_ and CH_4_ were exceeded together on 190 (8%) occasions. To consider an individual deployment, we illustrate all the sampled readings from the *location C* field deployment in Figure 10. In this case, the CO_2_ component exceeded the recommended limit [24] in 96.6% of the samples, while CH_4_ never exceeded the recommended limit [24] *i.e.*, 0% of the time. The average CO_2_ value recorded was 3.78%, which is 2.52 times above the regulatory limit of 1.5% v/v. The average CH_4_ value was 0.01% which is within the regulation limit of 1% v/v. It is worthwhile to note that, CO_2_ levels in soil/sub soil layer can naturally exceed 1.5% due to a number of external processes e.g., aerobic degradation of organic matter in soil, dissolution of CO_2_ from groundwater high in carbonate, microbial methane oxidation. Thus CO_2_ levels above 1.5% do not necessarily indicate landfill gas migration, however our methodology follows well established procedures and pre-existing monitoring routines by the EPA. An ideal solution would be to investigate typical background levels in the area being monitored, which are unaffected by the landfill. Furthermore, the levels quoted are limits for air; the borehole levels tell us the concentrations of these gases migrating within the landfill site that could be released into the air, and could be inherently dangerous if left uncontrolled [31].

Even considering this, observing the 7 month trend of sampled data, significant CO_2_ events were recorded around the 17th of March, 28th April, and 25th of September. Greenhouse gas emissions from landfills are inherently dynamic (especially during their initial phase) and events such as these can be attributed to a number of factors including: borehole proximity to the landfill, time of year, seal of the borehole well cap, water table, head-space, sample depth as well as human activities and extraction system failures/blockages. It is difficult to pinpoint the exact cause(s) of these events at this early stage in our investigations, but clearly, the availability of this type of information will open the way to gaining a fuller understanding of the dynamics of greenhouse gas generation in, and therefore more effective management of, landfill sites. This only strengthens the need for this type of real time monitoring technology. Finally, one important issue that arises from this data series is: how many of these events are missed by the current manual monitoring frequency of once per month. The next section explores this question.

### 4.4. Human Operator Error Simulation

**Figure 11.**
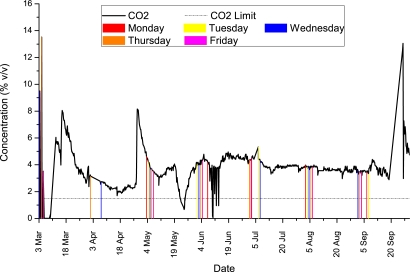
CO_2_ readings over a 7 month field deployment at *location C* between March 2010 and October 2010. Note that a simulated human operator sampling the landfill emissions on a particular first day or each month would miss a lot of events of interest e.g., the middle of March-2010, the end of April-2010, and the middle of September. The average error across each of the 5 days (Mon–Fri) would have been 17% in our field deployment at *location C*.

Considering Figure 11, in which we simulate a human operator taking a reading on a particular first day of each given month, it can be seen that many dynamic events would not be noticed particularly for the middle of March-2010, the end of April-2010, and the middle of September. For example if a human operator noted a reading on the first Monday of every month (at 12 noon), then there would be an average error of 7% (4.05% for CO_2_ *vs.* actual average reading of 3.78% from all sampled data points over 7 months). The first Tuesdays in our dataset would have yielded an average error of 11%, Wednesdays an error of 35%, Thursdays 33%, and Fridays an average error 0%. From these 5 scenarios alone (plus a visual inspection of Figure 11), it can be seen that there is a wide degree of error in selecting a manual rota for human operators to monitor overall landfill emissions.

### 4.5. Sampling Procedure Analysis

The sampling method used in this study has been developed on the back of detailed previous investigations into how best to sample the geochemical gas composition levels so that a representative and an accurate analysis is obtained [24,26,32]. These have shown that the sensors and sampling routine are not affected by other parameters such as flow rate or pressure, but by the sensing targets within the borehole. In addition, although this study focused on our national (Irish) acceptable gas emission levels of 1% for methane and 1.5% for carbon dioxide, *i.e.*, to reflect deployments locally, it should be noted that these acceptable levels are similar in many countries such as the United Kingdom [33], Canada [34], India [35], *etc*. with a similar recommended sampling frequency of once per month. It is also interesting to note that all have limited the methane level at 25% of its lower explosive limit *i.e.*, between 1% and 1.25% v/v.

**Figure 12.**
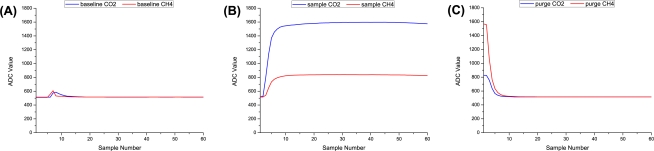
Profile of a typical 9 minutes *baseline* (A), *sample* (B), & *purge (C)* sampling stage, which is comprised of 180 CO_2_ & CH_4_ samples recorded every 3 seconds. This occurs in the order of 60× *baseline*, 60× *sample*, and 60× *purge* samples. Initially all 180 items were sampled, however after a close analysis of 10+ weeks of data, we have been able to minimise the length of this sampling procedure. This has a positive effect on battery power consumption.

To carry out our computational analysis we considered data from *location A* between 29th July 2009 and 9th October 2009. As described, our system firstly carried out a 3 minutes *baseline* stage, followed by an actual 3 minutes *sample* stage, and finally a 3 minutes *purge* stage (see Figure 12). We now discuss the CO_2_ and CH_4_ profiles associated with each of those stages. Note: all data presented here is from the full dataset downloaded from the field-deployed landfill units.

#### Baseline Stage

The average *baseline* profile (across the entire 143 recorded readings) is illustrated on the left hand side of Figure 12. Throughout our field deployment, the transmitted CO_2_ and CH_4_ *baseline* readings were calculated by taking the last 11 readings (33 seconds) of the full dataset (*i.e.*, after the sensors had time to warm up and just before the sampling stage) and calculating the average, however on post-analysis inspection of the baseline profile in Figure 12 it appeared we could get near that average by taking fewer samples. We then ran a software simulation program which went through 95 variations, on all 143 × 60 *baseline* readings. Our finding was that after 20 readings, and taking the average of the last 5 samples we then achieve a very low average reading error of 0.17% for CO_2_ and 0.62% for CH_4_, with an individual outlier worst case of 2.51% (CO_2_) and 2.57% (CH_4_) error in ADC reading, compared to the field-deployment implementation. This means that we have identified the point at which the sensors achieve a steady state response after a necessary and unavoidable warm up period. Overall, this represents a battery saving of over 60% for the *baseline* sampling stage alone.

#### Sample Stage

Next, the average *sample* profile (across the entire 143 recorded readings) is illustrated in the centre of Figure 12, for the chemical and physical sensors, respectively. Throughout our field deployments, the CO_2_ and CH_4_ composition levels were calculated by taking the maximum CO_2_, CH_4_, Humidity & Temperature readings (60 3-second samples taken in total). However, on inspection of the sample stage in Figure 12 it appeared that we could get those maximum values through taking less samples by visual inspection of the trends alone. We then ran a software simulation program which went through 95 variations, on all 143 × 60 *sample* readings. Our finding was that after 30 readings, and taking the average of the maximum sampled values, an average error of 0.72% (CO_2_), 0.34% (CH_4_), 0.07% (Humidity), and 0.007% (Temperature) is obtained, with an individual outlier worst case of 2.88% (CO_2_), 2.55% (CH_4_), 1.04% (Humidity), and 0.17% (Temperature) error in ADC reading, compared to the field-deployment implementation. This represents a potential battery reduction of 50% for the *sample* stage.

#### Purge Stage

Lastly, the average *purge* profile (across the entire 143 recorded readings) is illustrated on the right hand side of Figure 12. Throughout our field deployment, CO_2_ and CH_4_ levels were calculated by taking the minimum recorded readings (60 3-second samples taken in total), however on post-analysis inspection of the purge stage in Figure 12 it appeared we could get such a representative value by taking less samples. We then ran a software simulation program which went through 95 variations, on all 143 × 60 *purge* readings. Our finding was that after 20 readings, and taking the minimum recorded value we then get an average reading error of only 0.28% for CO_2_ and 0.12% for CH_4_, with an individual outlier worst case of 2.74% (CO_2_) and 0.78% (CH_4_) error in ADC reading, compared to the field-deployment implementation. This represents a battery saving of over 60% for the *purge* sampling stage.

So in summary, if we consider using a system as follows: 1 minute baseline; 1.5 minutes sample; and 1 minute purge, we would be within an average sample stage error of 0.72% (CO_2_), 0.34% (CH_4_), 0.07% (Humidity) and 0.007% (Temperature), with a worst case of 2.88% (CO_2_) in ADC readings. This would mean that our total field trial samples could be reduced from 10,010, as opposed to 25,740 which would represent a potential extension of battery life by 2.57 times.

#### Removal of Baseline & Purge Stages

As noted in Table 1 there was a period of time in our *location C* deployment where there was a 9 minutes monitoring period (with no baseline extraction beforehand or purge extraction afterwards). Comparing the very similar signatures of the sample stage in Figures 12 (*baseline + sample + purge*) and 13 (*sample only*), there is an indication that only the sample stage is needed. Carrying out a software optimisation of a *sample only* system, we found that just 30 (× 3 seconds) readings are required. The first 10 readings (*i.e.*, 30 seconds) are required for the sensors to heat up to a steady state and also for the pump to flush the sampling chamber in order to eliminate any ‘memory effects’ that may be present from the previous sampling stage.

#### Sampling Frequency

The initial use of this platform was to provide remote access to accurate data for enforcement purposes (EPA); four samples per day is a great improvement on one sample per month (EPA’s current procedure). It is also a balance between sampling rate and power demand we anticipate that the incorporation of the solar panel will enable the sampling frequency to be increased towards the levels required for more effective modelling of the site and optimisation of operation. In that respect, there may be more value from the monitoring of other targets such as pressure at higher frequencies as elevated levels of gases are often associated with blockages in the extraction system.

### 4.6. Lessons Learned

The evaluation procedure explored previously is not sufficiently comprehensive at this stage to make a definite case to drop the baseline and purge stages. The most effective way to do this would be to run the 2 systems in parallel and verify that both are analysing the same landfill gas. However, from all the data and experience available to us, we feel that a *sample only* system is the best approach to take in future deployments. We recommend a sampling stage as follows (at least 4 times per day):

1. Allow 30 seconds for sensors to warm up (no sampling required)
2. Sample every 3 seconds for the next 90 seconds (CO_2_, CH_4_, Humidity, Temperature)
3. Stop if 5 consecutive readings report the same values
4. Record the maximum reported values from step 2 for CO_2_, CH_4_, Temperature, and Humidity

Such a system, compared to that deployed in our field trials (9 minutes samples with *baseline, sample,* and *purge* stages), would offer a number of advantages:

- No valves required, so less mechanical complexity and cost, and increased reliability and battery life.
- Total sample time reduced from 9 minutes to 1 m 45 seconds A saving of 80% in active power consumption, and potentially increasing battery field lifetime from 6 weeks to approximately 30 weeks; this figure does not factor in power savings achieved through not having to actuate valves.
- Reduced manufacturing costs and increased battery life and reliability, as switching valves are no longer required.

### 4.7. Future Work

#### Communications

At present we have achieved remote data retrieval through compiling a statistical representation of the data and by transmitting to the base station by means of SMS. The SMS text structure was formed so that it could be readily interpreted by a human observer. Although this option was beneficial at early stages of the project, we have since progressed to a complete data orientated formulation where human observations are now at the visualisation end (see Figure 6). As a result, one can retrieve a richer sensor data-set by introducing encoding schemes (such as Huffman or Arithmetic) to compress the data and ultimately retrieve more information per transmission. This will reduce the transmission frequency resulting in lower cost and power use. Furthermore, this can potentially result in retrieval of fully recorded datasets via SMS without the user physically being present.

Alternatively when we deploy multiple units on a site an additional communications layer should be considered, whereby the units will be equipped with low power, short range radio transceivers (such as Zigbee). Then each sensor would report all its findings to a central communications gateway over a star/mesh/bus wireless sensor network as outlined by a recent survey [36]. We foresee that the communications gateway will relay all data to the base-station via GPRS/3G on sites without any local access points. On the other hand, many active sites have a nearby workplace with internet capability where one can potentially take advantage of new generation technologies such as WiMAX [37]. This strategy will give a new layer of scalability to the sensing structure and allow many other plug and play sensing nodes to be added (such as more sensed locations, wider range of gases e.g., H_2_S, gas pressure monitoring *etc*.).

Ultimately, the chosen communications method will depend on the layout of the site and also on the number of nodes needed. At this stage in our deployments, SMS communications has been found to be sufficient to explore this application principle.

#### Adaptive Sampling

Borehole measurements typically involve monitoring the sensor output until a steady state signal is achieved for a representative gas sample. Many factors have been found to affect this and have been listed earlier in Section 4.3. A subset of these has already been explored in a previous study [26] and to accommodate these factors, we have chosen to sample for a period of 3 minutes. However, there are disadvantages to this approach with the primary drawback being unnecessary power usage on wells that generate a steady state signal relatively quickly. This ultimately reduces the lifetime of the device in the field.

Although we have addressed this issue earlier (Section 4.5), we foresee a further extension of an overall adaptive sampling technique including a varied sampling cycle frequency, *i.e.*, a fixed frequency of 2 times per day may be too high for some sites and too low for others. We foresee the application of computer classification techniques to adaptively select the optimum sampling frequency, based on previous reported gas concentrations and battery capacity at the time.

#### Energy Reduction/Harvesting

It has been determined earlier (Section 4.2) that the system can function autonomously for circa 4.5 weeks using the current power source. Although this was acceptable for our purposes, it is desirable to maximise the functional lifetime of the system (where possible) in the environment. The principle reason for this is that the cost of maintenance alone (for battery changes and the human resources required to change them) can be substantial, especially with multiple sites. A future option to explore is the harvesting of energy through using devices such as solar panels in the first instance. This is a crucial limiting factor, as scalability depends on sensors being able to meet their operational energy requirements from integrated energy generation capabilities. Our most recent efforts have made significant headway with the integration of a solar panel and anticipate that we will be deploying systems in the field in the near future for evaluation. Laboratory data suggests that standard panels will be able to meet the power demand of the platform and dramatically extend its operational lifetime.

#### Integration of Other Sensors

It should be noted that CO_2_ and CH_4_ were the primary sensing targets as identified by the EPA; they are also accessible via IR sensors that are very reliable and as we have demonstrated, suitable for long term autonomous deployment (greater than 1 year) with platforms that are relatively low cost. Inclusion of additional targets means complicating the sensing platform, potentially driving up the cost significantly, and reducing the capacity to function autonomously, which is directly against the goal of the project. So while we appreciate that additional targets are important, we had to strike a balance between long term viability and number of targets.

Conceptually, our setup can accommodate many more sensor types (with minimum alterations to the gateway platform) and also many more gateway platforms. Also, individual components such as the communications module can be swapped out very easily to accommodate other standards.

Recently, we have equipped a landfill unit with other chemical gas sensors to monitor levels of ammonia (NH_3_) and hydrogen sulphide (H_2_S) by introducing simple signal conditioning circuitry to the system, and we are ready to interface the system to the portal page once a suitable location has been found. In addition, we have already expanded the sensor server and web visualisation interface to include real-time monitoring of phosphate (PO_4_^3−^) in river waters, carbon monoxide (CO) in car parks, domestic carbon emissions and pressure of extraction systems on landfill sites [38].

#### Future Deployments

This study has provided the means to fully equip landfill sites with multiple analyser platforms to detect landfill gas migration and/or surface emissions. In the first instance, our aim is to increase production of the systems and launch a case study on a young, active landfill site. This will allow us to harvest data across an entire site and over a long period of time with an end goal to apply software modelling techniques. Ultimately this may provide a real enhancement of landfill management capabilities, e.g., through the development of early warning systems. Furthermore, with such a deployment, we may be able to identify many more aspects of landfill dynamics such as seasonal effects, tears in liners during installation, blocked pipes *etc*.

## 5. Conclusions

We have successfully realised and validated a platform for real time monitoring of landfill subsurface migration gases. Our system incorporates sensing of carbon dioxide and methane emissions at landfill sites, GSM communications to a “cloud database” and an on-line visualisation element to deliver near real time data to users in an easy to interpret format. This system has been successfully deployed in field-tests over a 16 month period, with 3 separate devices running concurrently across multiple locations towards the end of the trial generating 2,445,300 *in-situ* measurements of gas concentrations during this time.

Through post analysis of the data gathered over a 16 month period, we have identified further improvements that we can make to the system to reduce the cost and power consumption. The advantages of our remote monitoring system is that it consistently gathers data at a much higher granularity than the current manual sampling regime thereby reducing the risk of missing events, in addition to reducing the possibilities for human operator error. From experience “at the coalface” through long-term field trials, and through involvement with relevant environmental and industrial stakeholders, our system has evolved to the stage where we are confident that it is now possible for authorities to complement their manual monitoring with a much more rich stream of data.

Finally, one conclusion of particular interest is that we have shown that this approach, with much higher sampling frequency, opens up a rich source of new environmental information about the dynamics of landfill gas generation and migration, that can ultimately lead to a better understanding of the processes underlying “events” (rapid increases in emissions), and therefore, more effective management of these facilities.

In addition, recent events have shown that catastrophes can and do occur at landfill sites, *i.e.*, a massive underground fire erupting at a nearby 50-acre site in Co Kildare, Ireland. This has had an overall negative impact in many ways such as health risks to thousands of nearby residence through pollution of the surrounding air, evacuations and it has been estimated that it will cost more than 30 million euros to recover from this disaster [31].

It is clear that we must rethink our approach to effectively and efficiently managing our domestic waste responsibilities via land filling, to one where we start to utilise the extensive capabilities of the sensor research community. The approach proposed in this study aims at providing a means to that end.

## Figures and Tables

**Figure 13.**
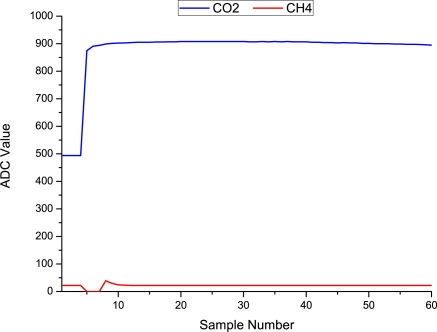
Profile of our *“sample only”* system, note the same signature as Figure 12, which possibly indicates that only the *sample* stage is needed to measure the air emissions at a given landfill site.

**Table 1.** Field deployment data gathered over a 16 month period. For *location C* a 9 minutes *sample only* approach was taken, as opposed to *baseline + sample + purge.*

Location	Start Time	End Time	Num Data Readings	Sampling Rate	CO_2_ Avg %	CH_4_ Avg %	CO_2_ Limit Exceeded	CH_4_ Limit Exceeded	CO_2_ & CH_4_ Exceeded
A	28-May-09	08-Oct-09	255	2×/day	7.71	13.70	185	133	133
B	13-Aug-09	08-Oct-09	113	2×/day	3.23	0.13	77	0	0
A	20-Nov-09	28-Dec-09	77	2×/day	5.99	0.85	58	10	10
C^*^	03-Mar-10	07-Sep-10	764	4×/day	3.78	0.09	738	0	0
A	10-Mar-10	07-Sep-10	768	4×/day	1.52	0.30	243	47	47
B	24-May-10	07-Sep-10	446	4×/day	1.48	0.02	189	0	0

SUM			2,403				1,490	190	190
